# The price of being late: short- and long-term consequences of a delayed migration timing

**DOI:** 10.1098/rspb.2023.1268

**Published:** 2023-07-26

**Authors:** Iris D. Bontekoe, Roland Hilgartner, Wolfgang Fiedler, Andrea Flack

**Affiliations:** ^1^ Department of Migration, Max Planck Institute of Animal Behavior, 78315 Radolfzell, Germany; ^2^ Collective Migration Group, Max Planck Institute of Animal Behavior, 78467 Konstanz, Germany; ^3^ Department of Biology, University of Konstanz, 78457 Konstanz, Germany; ^4^ Affenberg Salem, 88682 Salem, Germany; ^5^ Centre for the Advanced Study of Collective Behaviour, University of Konstanz, 78468 Konstanz, Germany

**Keywords:** migration, social information, time–energy trade-off, movement ecology, white stork, experiment

## Abstract

Choosing the right migration timing is critical for migrants because conditions encountered *en route* influence movement costs, survival, and, in social migrants, the availability of social information. Depending on lifetime stages, individuals may migrate at different times due to diverging constraints, affecting the composition of migration groups. To examine the consequences of a delayed migration timing, we artificially delayed the migration of juvenile white storks (*Ciconia ciconia*) and thereby altered their physical and social environment. Using nearly continuous 1 Hz GPS trajectories, we examined their migration behaviour, ranging from sub-second level performance to global long-distance movement, in relation to two control groups. We found that delayed storks experienced suboptimal soaring conditions, but better wind support and thereby achieved higher flight speeds than control storks. Delayed storks had a lower mortality rate than the control storks and wintered closer to the breeding area. In fact, none of the delayed storks reached the traditional African wintering areas. Thus, our results show that juvenile storks can survive migrating at the ‘wrong’ time. However, this had long-term consequences on migration decisions. We suggest that, when timing their migration, storks balance not just energy and time, but also the availability of social information.

## 1. Background

Seasonal migrations allow animals to experience suitable conditions across seasons [1]. When performing these journeys, migrants have various costs and benefits that depend on their migratory decisions. To decide when and where to go, some species genetically inherit migration patterns (e.g. [2,3]), while others use social learning [4,5] where juveniles learn migration behaviour from one or both of their parents (e.g. [6,7]). In contrast to following their parents, juveniles can also migrate with other conspecifics to benefit from travelling with unrelated individuals and similarly aged peers [8]. While parents have an interest in transmitting optimal migration patterns to their young [9,10], this may not be true for non-family members. Unrelated individuals may not slow down to maintain group cohesion or to decrease juvenile energy expenditure, like it has been shown for parent–offspring pairs [7]. In such cases, the composition of the migration group may affect juvenile performance by influencing migration costs and the amount of migration information that is available. More specifically, the ratio of inexperienced to experienced individuals in a migratory group may affect broad-scale migration features like routes and destinations, but also migration speed and costs. Thus, considering the impact of social dynamics (e.g. group cohesion and composition) may be essential when it comes to studying migration behaviour and its development.

Many migratory birds are known for their tendencies to migrate in large flocks composed of related and unrelated individuals [11], offering suitable study systems for exploring the effect of group composition. The features of migration flocks may depend strongly on migration timing, since birds of different ages often migrate asynchronously due to changing trade-offs and pressures during different lifetime stages [12]. For adults, autumn migration timing is affected by breeding behaviour [13,14] and the subsequent pre-migration and moulting period [12]. Breeding behaviour of (successfully) reproducing individuals may therefore constrain early autumn departures, leading to later migrations. While the need to breed likely pushes adults towards a more time-efficient migration, immatures may need an exploratory phase in early life, during which they gain experiences that benefit the establishment and refinement of their migration behaviour [15,16]. This could be expressed as juvenile birds taking longer [17,18] or seemingly disadvantageous routes compared to adults from the same population [19,20]. However, the ability to detect and explore novel habitats and regions may rely strongly on the availability of social information during the different migration stages [21,22] and therefore on the right migration timing.

Adjusting migration timing also affects migration speed and migration costs as these are determined by local environmental conditions that deteriorate with the advancing season. Further, immature and adult individuals also exhibit different abilities to exploit environmental conditions for cost-efficient movements or energy intake. Juveniles often depart under less beneficial wind conditions than adults [23], and learning to cope with crosswinds may take several years [24,25]. In addition, juveniles are often slower fliers than adults [26] and generally fly less efficiently [27]. Instead of using flapping flight, soaring–gliding birds have evolved to exploit rising air to fly in an energy saving way [28]. Juveniles often gradually increase the amount of soaring–gliding flight [29]. Flying in flocks with adults can help juveniles to locate thermals (columns of rising warm air) [30], however, juveniles still spend more energy by flapping more than adults [30,31]. Finally, while wind support can be very beneficial for migrants by increasing speed or reducing costs [32,33], flying under adverse wind conditions can increase mortality [34]. Thus, choosing a suboptimal migration timing does not just affect the costs of the journey but also the migratory community that provides guidance and learning opportunities.

In this study, we investigate the consequences of a delayed migration timing on juvenile birds in a social migrant with age-related differences in migration timing. By doing this, we aim to quantify the effect of an altered physical and social environment during migration in terms of movement costs and migration patterns. To explore this, we used the white stork (*Ciconia ciconia*; hereafter stork) as a model species. Storks are long-distance, soaring–gliding migrants that migrate in mixed juvenile-adult flocks that can be as large as several hundreds of individuals. It is often stated that juvenile storks learn their migration route from older birds [35], however adult storks usually migrate later than their offspring [31]. Due to this temporal variation between juveniles and adults, it is reasonable to expect that early migrating juveniles migrate with fellow juveniles and non-breeding birds, while late juveniles encounter more older and more experienced adults. Here we delayed the timing of migration of juvenile white storks, causing them to experience a different physical and social environment than they normally would. We recorded their migration in high detail and compared it to two control groups. We predict that delayed juvenile storks have a higher movement activity (i.e. flapping behaviour measured using three-axial accelerometer data) during migration, because they experience less suitable thermalling conditions. Delayed storks may have to use more flapping flight in order to prevent grounding, or to cope with faster flight speeds of their older and more experienced flock mates. In addition, overall migration patterns and survival of the delayed juveniles may be different because of the altered social environment.

## 2. Methods

### (a) Study design

To investigate the effect of migration timing, we compared the migration and flight performance of juvenile white storks from three GPS-tracked study groups. First, the ‘naturally timed storks' migrated under regular conditions: 40 juvenile storks were GPS-tagged in 2020 throughout southwestern Germany. These storks were returned to their nest after receiving their GPS device. Second, the ‘control storks' were taken into an aviary of a care centre station near Freiburg (Germany; 48.079169°N, 7.812155°E) shortly after fledging but were released mid-August to be able to migrate at times when migration naturally occurs. Control storks were tagged and released in 2019 (9 storks) and 2020 (10 storks). Despite the release in mid-August, the control storks migrated on average 12 days later than the naturally timed storks that were tagged in the same area (see electronic supplementary material, figure S1). Third, ‘delayed storks' were taken out of their nest before fledging and retained in an aviary until mid-September (see details below). All storks were outfitted with leg bands for individual identification and with solar-powered GPRS-GPS-ACC loggers (e-obs GmbH, Germany). Loggers weigh 42 g and were attached using a wing harness made of Teflon-nylon and a metal ring (approx. 12 grams). In total, the tag and harness weigh less than 2% of the storks' body weight. Tags have been successfully used for other tracking studies on storks [31,36,37] and recorded the birds’ GPS positions every 15 min between 2.00 and 20.00 GMT (4.00–22.00 local time). Depending on the speed of the storks, such a position was either a single position (when stationary) or a 10-min burst at 1 Hz frequency (when in flight). Directly after the GPS position, tri-axial accelerometer (ACC) data were collected in four-second bursts at 10 Hz. Data were retrieved using a hand-held base station or via the phone network (when available).

### (b) Delayed release

In two years (2019 and 2020), a total of 40 juvenile storks (20 per year) were taken out of their nest about one week before their estimated fledging time (age: approx. 7 weeks) from the breeding colony at Affenberg Salem (Germany; 47.762240°N, 9.245043°E). Storks were kept in an aviary of about 30 × 12 × 4 m that was located next to the breeding colony. Since the top of the aviary was only enclosed by nets, the delayed storks were able to see and hear conspecifics and they could observe other birds soaring in thermals in the proximity of the aviary. However, except for short-distance flapping flights, the delayed storks did not have the opportunity to fly in the aviary. Delayed storks were released in mid-September of their corresponding years, approximately one month after naturally migrating juvenile storks migrated. In 2019, 17 of them were released near Leibertingen (Germany; 48.033043°N, 9.004508°E) and 3 near Winterspüren (Germany; 47.851944°N, 9.072189°E). In 2020, all 20 storks were released at the same location near Leibertingen. Release locations were chosen because they had a low stork breeding abundance, in an attempt to prevent the released groups from splitting up immediately and to avoid contact with untagged storks. As far as possible, the flocks were followed over land by human observers until they joined other storks. Once the delayed storks joined other storks, efforts were made to read the leg bands of the entire flocks to determine the age and origin of individuals. The large majority of the storks that our delayed storks encountered was at least 1 year old (see electronic supplementary material, table S1). If storks died, due to high first-year mortality [38], we located the carcasses in the field to retrieve the tag and to determine the cause of death.

### (c) Environmental variables

GPS data were annotated with environmental variables to assess flight performance relative to the experienced conditions. Weather variables were obtained from Movebank [39,40] using the bilinear interpolation within the Env-DATA tool [41]. Elevation measures (in metres [42]) were used to calculate flight altitude from the GPS recorded height above ellipsoid. Atmospheric data came from the European Centre for Medium-Range Weather Forecasts (ECMWF; www.ecmwf.int). U (east-west) and V (north-south) wind velocity (in m s^−1^ [43]) at pressure level (i.e. at the atmospheric pressure level that corresponds to the altitude of the bird) were downloaded and used to calculated wind speed and direction. Wind support and airspeed were then calculated following [44]. To test for differences in weather patterns between years, we annotated the tracks of three delayed storks and three naturally timed storks within a representative migration segment (see below) with U and V wind velocity, air temperature (in K at 2 m above the ground [43]) and boundary layer height (in metres [43]). Each of these six tracks was annotated with environmental data for each date across the entire range of dates between 29 July (first date a stork entered the segment) and 25 September (last date a stork left the segment) in 2019 and 2020. Since we annotated the tracks with weather data from multiple days, we believe that using a subset of storks results in a representative view of weather conditions that were encountered by all storks.

### (d) Data processing

Few delayed storks (2019: 2 out of 20, 2020: 5 out of 20) were excluded from the analyses, except for determining overall mortality, because they died near the release site. In 2019, a delayed stork was weakened and taken back into the aviary. In 2020, one of the control storks was taken to a care centre in France during migration. These storks were considered as if they had died at the moment they were captured. As a measure for overall survival, we calculated the proportion of storks that survived during their first year (up to 30 June in the year after tagging).

Total migration distance was calculated as the geodesic distance between the release location (control and delayed storks) or the first location on 1 July (for naturally timed storks) and the location of the most southern latitude reached by each stork, using the package geopy [45]. We defined a few thresholds to quantify migration behaviour. To classify storks as migratory, we used a threshold of a total migration distance larger than 200 km. If a stork had a migration distance larger than 3000 km, it was considered to have reached sub-Saharan Africa. Storks that reached their most southern latitude less than three days before death or within five days after the release were excluded from the analyses since it is unclear whether they had finished migrating.

For the fine scale analyses, we used data from one representative segment of the migration. This segment overlapped between all migrating storks and ranged from southern Germany (47.5°N) to southern France (44°N). We used this segment to avoid extreme differences in environmental conditions, i.e. comparing storks migrating in northern Europe with those in sub-Saharan Africa. While this controls for geographical location, it cannot completely control for the differences in encountered environmental conditions that occurred because storks migrated at different times. Delayed storks entered the segment 5 ± 1 (mean ± s.d.) days and control storks 10 ± 3 days after their respective releases (see electronic supplementary material, figure S2). The first GPS burst that ended in the segment defined the time an individual entered the segment; the first GPS burst that ended south of the segment defined the leaving time. Only migration days (daily displacement between first and last location larger than 50 km) were used for analyses of flight behaviour. Short flights (e.g. flights between foraging and night locations) in the morning and evening were excluded from the data. Days on which storks displaced less than 50 km between the first and last location of the day counted as stopover days. Only storks that entered the segment in the north and left the segment in the south were included in the analyses. This led to 36 naturally timed storks, 17 control storks and 15 delayed storks.

We measured several migration and flight features, and weather variables to detect potential differences between the study groups. Route straightness is the straight-line distance between entering and leaving the segment divided by the cumulative distance between all locations within the segment. Daylength, was calculated as the time in hours between sunrise at the first location of the day and sunset at the last location of the day using the package astral [46]. Daily flight time is the time between the start and the end of the main migration flight on a given day, excluding short flights in the morning and evening. Daily distances (km) were calculated as geodesic distance between first and last locations of a given day. Cross-country speed (km h^−1^) is the geodesic distance covered in a migration flight divided by the flight duration on a given day. For testing the effect of wind on cross-country speed, we calculated the average U and V wind experienced during daily migration flights. We also calculated wind support in the direction of the straight-line distance between the first and last location of the daily migration flight following [44].

Climbing rates were calculated in metres per second using the difference in height above ellipsoid between measurements within each GPS burst. GPS locations were assigned to flight bouts whenever the smoothed ground speed was above or equal to 2.5 m s^−1^. Climbing and gliding bouts were classified based on the smoothed climbing rate (above 0.2 m s^−1^: climbing, below 0 m s^−1^: gliding) during flight bouts. Flight, climbing, and gliding bouts had to be at least 15 s long, with a maximum interruption of 5 s during which the stork performed a different behaviour. Smoothed ground speeds and smoothed climbing rates were calculated using a running window length of 15 s. Climbing rate and sinking speed are the average raw climbing rate during climbing and gliding bouts, respectively. The altitude at which storks left thermals corresponds to the altitude at the end of climbing segments that did not end in the last 5 s of a GPS burst.

Overall dynamic body acceleration (ODBA) was calculated for each ACC burst. First, the ACC measurements were converted to m s^−2^. Then, for each axis, we calculated the absolute deviation between each measured value and the axis-mean. To get ODBA, we summed the mean of the deviations of each axis. Climbing or gliding behaviour (see above) was assigned to each burst based on the behaviour of the stork at the end of the preceding GPS burst.

Data were processed in Python 3.8.5 [47].

### (e) Statistical analyses

We compared various migration and flight features, and weather variables between the study groups. Unless stated otherwise, all statistical models had study group (i.e. naturally timed, control, delayed) as explanatory variable to test for the consequences of the altered migration timing. When we had repeated measures for individuals (e.g. daily distance and climbing rate), we used linear mixed-effects model (LMM; package lmerTest [48]) ANOVAs. All LMM-ANOVAs were performed with the Kenward-Roger method (see [49]) and with individual ID as random intercept. When we had one measure per individual (e.g. migration distance and timing), we used linear model (LM) ANOVAs. Whenever an LM(M) indicated a significant effect of study group (*p* < 0.05), we additionally performed a *post-hoc* Tukey HSD test (Tukey test; package multcomp: [50]) to find out which study groups differed from each other.

To test for differences in migration propensity (migratory or resident) and in the tendency to cross the Sahara Desert (yes or no) between the three study groups, we used a Fisher's exact test of independence followed by a *post-hoc* pairwise Fisher's test (package rstatix [51]) with a Benjamini-Hochberg FDR *p*-value correction (see [52]). To explore differences in migration distance between groups, we used an LM-ANOVA. To test for differences in wintering latitude for the first migration-year and for the second migration-year, we performed separate LM-ANOVAs. We also used LM-ANOVAs for the first and second year separately to assess differences in migration timing between the study groups.

To test for differences in the total number of days in the segment and the number of stopover days in the segment between the study groups, we used a Kruskal–Wallis rank sum test followed by a pairwise Wilcoxon rank sum test with a Benjamini-Hochberg FDR *p*-value correction [52]. We used an LM-ANOVA to test for differences in route straightness between the study groups. We compared differences in experienced daylength, differences in daily flight time, distance covered per day, and cross-country speed between groups using separate LMM-ANOVAs. To investigate the influence of wind on cross-country speed, we compared LMMs including (1) study group and wind support, (2) study group, and (3) wind support using Akaike's information criterion [53]. In these models, we included repeated measures (one per day in the segment) per individual.

Differences in climbing rates and in the altitude at which storks left thermals were assessed using LMM-ANOVAs. Both response variables were square root transformed to improve the model fit. To test for differences in ground speed during gliding, we used an LMM-ANOVA with log-transformed data to improve the model fit. We used LMM-ANOVAs to test for differences in airspeed, wind support during gliding, and sinking speed. In these models, we included repeated measures (multiple GPS bursts) per day and individual. To test for differences in ODBA, we used an LMM-ANOVA with log-transformed data to better fit the model assumption of normality. To test for a trend in ODBA over time, we fitted an LMM-ANOVA with the log-transformed ODBA of naturally timed storks (i.e. the group that represents the naturally migrating stork population) as response variable, day of the year as predictor variable and individual ID as random intercept. In these models, we included repeated measures (multiple ACC bursts) per day and individual.

Statistical analyses were performed in R v. 4.1.2 [54].

## 3. Results

We compared the migratory behaviour of juvenile white storks from three different groups (naturally timed storks, control storks and delayed storks; see Methods). Our analyses ranged from the broad, continental scale to fine-scale flight performance.

### (a) Routes, destinations and survival

**Figure 1.**
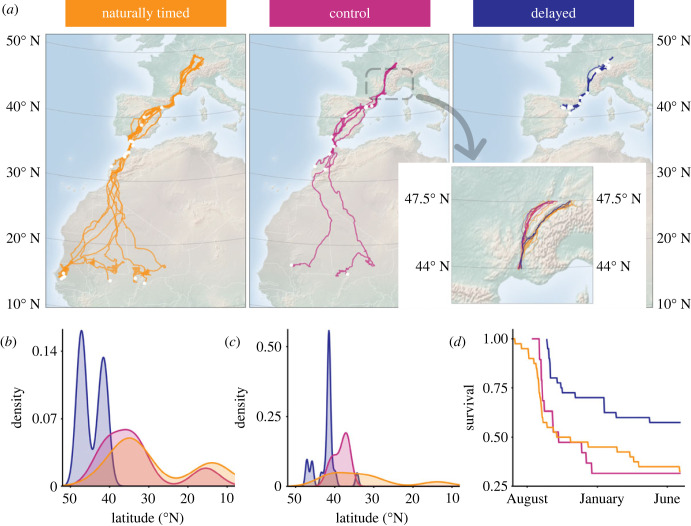
Differences in migration patterns and survival between naturally timed (orange), control (pink) and delayed (blue) storks. (*a*) Migration tracks from each of the three study groups (naturally timed: 23 individuals; control: 12 individuals; delayed: 32 individuals). White dots represent the location where the stork reached its most southern latitude. Inset: the migration segment. (*b*) Probability densities of wintering latitude for individuals from all three groups in their first migration year (naturally timed: 23 individuals; control: 12 individuals; delayed: 32 individuals), and (*c*) in their second migration year (naturally timed: 13 individuals; control: 5 individuals; delayed: 21 individuals). Wintering latitude is defined as the most southern latitude of each individual in the given year. (*c*) Survival (proportion of individuals in each group that were alive) against time of the year. For naturally timed storks (40 individuals), survival is shown from 1 June. For control (19 individuals) and delayed (40 individuals) storks, survival is only shown from their release dates. Probability densities are kernel density estimates based on histograms of the raw data, also referred to as smoothed histograms.

Delayed storks had a low migration propensity with only 15 out of 32 delayed storks migrating (2019: 14 out of 17; 2020: 1 out of 15). By contrast, all of the naturally timed and control storks migrated. The difference in migration propensity between delayed and naturally timed (Fisher's exact test, *p* < 0.001, *n* = 67; *post-hoc* pairwise Fisher's test, adjusted *p* < 0.001) and control (*post-hoc* pairwise Fisher's test, adjusted *p* < 0.005) storks is significant. The 15 delayed storks that did migrate had a shorter migration distance (960 ± 112 km; figure 1*a*) compared to the 23 naturally timed (2615 ± 1176; LM, F_2,47_ = 13.812, *p* < 0.001, *n* = 50; Tukey test, *p* < 0.001), and 12 control storks (1979 ± 1032; Tukey test, *p* < 0.05). Delayed storks only reached southern France or northern Spain (figure 1*a*), although the migration season was still ongoing (see electronic supplementary material, figure S3) and thermalling conditions would have allowed them to continue migrating (see electronic supplementary material, figure S4). The naturally timed storks migrated at least to mid-Spain or Morocco while 30.4% (7 out of 23 individuals) continued migrating to reach the traditional sub-Saharan wintering grounds (figure 1*a*). From the control storks, the majority migrated to southern Spain or Morocco, while 16.7% (2 out of 12 individuals) continued to the sub-Saharan wintering grounds. Significantly fewer delayed storks crossed the Sahara Desert compared to naturally timed storks (Fisher's exact test, *p* < 0.005, *n* = 67; *post-hoc* pairwise Fisher's test, adjusted *p* < 0.005), but there was no difference between any of the other study groups (*post-hoc* pairwise Fisher's test, adjusted *p* > 0.1). These differences in wintering latitude between the groups even persisted to the second year, despite migration timing becoming normal. In their first year, delayed storks wintered farther north than naturally timed (figure 1*b*; LM, *F*_2,64_ = 32.254, *p* < 0.001, *n* = 67; Tukey test, *p* < 0.001) and control storks (Tukey test, *p* < 0.001). In their second year, delayed storks also wintered farther north than naturally timed storks (figure 1*c*; LM, *F*_2,36_ = 9.937, *p* < 0.001, *n* = 39; Tukey test, *p* < 0.001). Groups differed in their migration timing (i.e. the first date individuals entered the segment) in the first year (electronic supplementary material, figure S5; LM, *F*_2,66_ = 118.967, *p* < 0.001, *n* = 69; Tukey test, *p* < 0.001 between all groups), but not in their second year (electronic supplementary material, figure S5; LM, *F*_2,25_ = 0.667, *p* > 0.5, *n* = 28). This means that a delayed migration in the first year had long-lasting consequences.

During their first year, delayed storks had a higher survival (23 out of 40) than naturally timed (13 out of 40) and control storks (6 out of 19). The majority of the naturally timed and control storks died during the southward migration period (figure 1*d*). Three non-migrating delayed storks died mid-January during a period with heavy snow in central Europe. Although we closely monitored all released storks, movement and acceleration patterns did not indicate these casualties upfront.

### (b) Duration and speed

**Figure 2.**
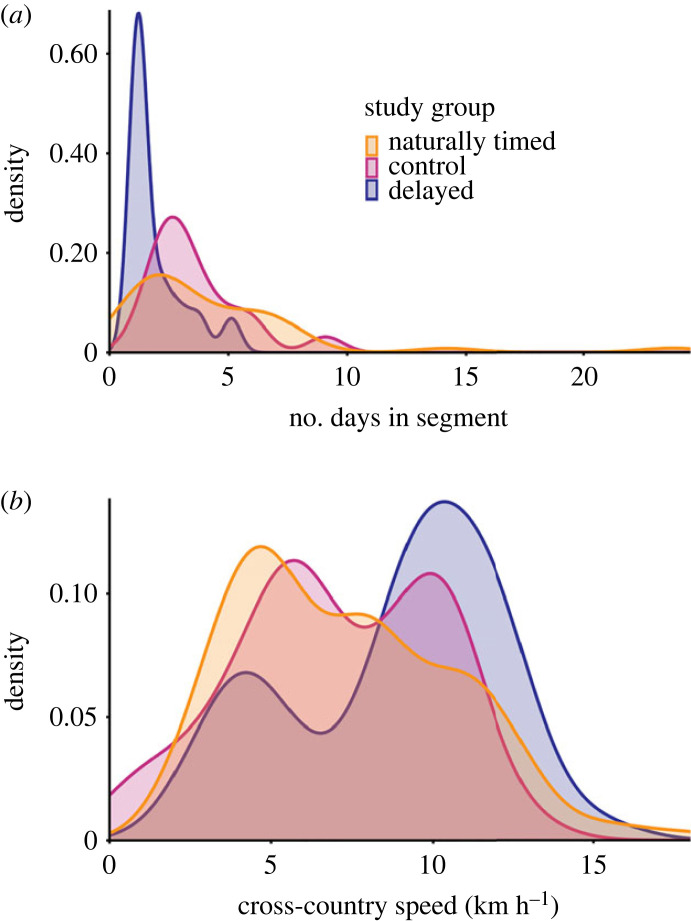
Differences in migration speed between naturally timed (orange, 36 individuals), control (pink, 17 individuals) and delayed (blue, 15 individuals) storks. (*a*) Probability densities of the number of days that individuals from each study group took to cover the migration segment. (*b*) Probability densities of cross-country speed per study group. Probability densities are kernel density estimates based on histograms of the raw data.

To understand fine-scale migration and flight patterns, we only used data of a migration segment ranging from southern Germany to southern France to reduce effects caused by differences in geographical locations between groups. Only individuals that covered the entire segment are included in the analyses here (naturally timed: 36 individuals, control: 17 individuals, delayed: 15 individuals). The time it took the naturally timed storks to cover this segment ranged from 1.0 to 23.8 days (median: 3.0 days). There was less variation for the control storks (1.2–9.1 days, median: 3.1) and delayed storks (1.2–5.1 days, median: 1.2). Delayed storks took fewer days to migrate from southern Germany to southern France (the segment) than naturally timed storks (figure 2*a*; Kruskal–Wallis rank sum test, chi-squared = 7.869, df = 2, *p* < 0.05, *n* = 68; pairwise Wilcoxon rank sum test, *p* < 0.05) and control storks (pairwise Wilcoxon rank sum test, *p* < 0.01). There was no difference between the number of days that control and naturally timed storks spent in the segment (pairwise Wilcoxon rank sum test, *p* > 0.5). The differences in duration corresponded to differences in the number of stopover days. While covering the segment delayed storks took fewer stopover days than naturally timed (Kruskal–Wallis rank sum test, chi-squared = 9.420, df = 2, *p* < 0.01, *n* = 68; pairwise Wilcoxon rank sum test, *p* < 0.05) and control storks (pairwise Wilcoxon rank sum test, *p* < 0.01).

There were no significant differences in route straightness between the groups (LM, *F*_2,65_ = 2.763, *p* = 0.071, *n* = 68), meaning that there was no effect of migration timing on straightness within the segment. Despite differences in daylength (LMM, *F*_2,65.77_ = 110.855, *p* < 0.001, *n* = 210; Tukey test, *p* < 0.005 between all groups), there was no difference in the daily flight time (LMM, *F*_2,64.62_ = 0.166, *p* > 0.5, *n* = 210) or distance covered per day (LMM, *F*_2,64.62_ = 2.538, *p* = 0.087, *n* = 210) between the groups. Delayed storks had a higher daily cross-country speed (8.83 ± 3.20 m s^−1^) than naturally timed (7.30 ± 3.33 m s^−1^; figure 2*b*; LMM, *F*_2,64.62_ = 4.018, *p* < 0.05, *n* = 210; Tukey test, *p* < 0.05) and control storks (7.01 ± 3.08 m s^−1^; Tukey test, *p* < 0.05), meaning that delayed storks covered distance faster on a daily basis. There was no difference in daily cross-country speed between naturally timed and control storks (Tukey test, *p* > 0.5). To investigate this difference in cross-country speed further, we added wind support to the model and compared AIC-values. The model with wind support had a lower AIC value (*Δ*AIC > 2) than both the model with wind and study group and the model with only study group as predictor variable. This indicates that differences in daily cross-country speed between the groups could be explained by differences in wind support early and late in the season. However, since we only examined the wind patterns during our study period (2019 and 2020; see electronic supplementary material, figure S6), it remains unclear whether beneficial wind support later in the season is a general pattern occurring every year.

### (c) Environment and costs

**Figure 3.**
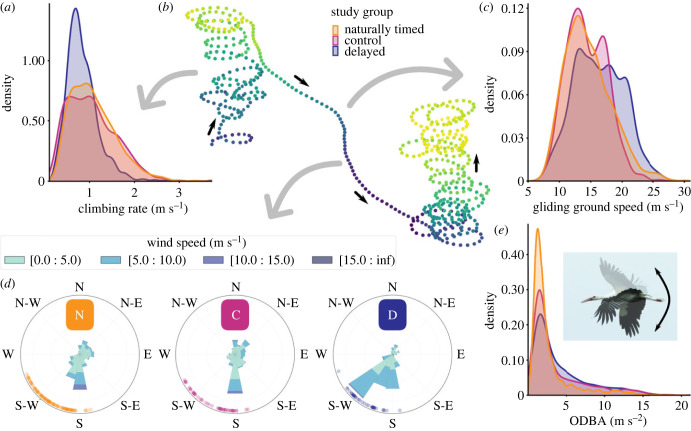
Differences in fine-scale flight performance between naturally timed (orange, 36 individuals), control (pink, 17 individuals) and delayed (blue, 15 individuals) storks. (*a*) Probability densities of climbing rates within the migration segment for each of the study groups. (*b*) Visualization of one 10-minute GPS-burst, consisting of two thermalling bouts with a gliding bout in between. Colours represent altitudes, ranging from blue (low altitudes) to yellow (high altitudes). (*c*) Probability densities of the ground speed during gliding bouts within the migration segment, for each of the study groups. (*d*) Windrose plots of experienced wind speed and direction during gliding bouts within the migration segment for each of the three study groups (N: naturally timed, C: control, D: delayed). Dots indicate the daily average heading of individuals during gliding bouts. (*e*) Probability densities of ODBA, an indication for the amount of flapping flight, for each of the study groups. Probability densities are kernel density estimates based on histograms of the raw data. The stork photo is taken by Iris Bontekoe.

Naturally timed storks and control storks had an overall higher climbing rate within the examined migration segment than delayed storks (figure 3*a,b*; LMM, *F*_2,65.36_ = 12.130, *p* < 0.001, *n* = 4410; Tukey test, *p* < 0.005). Also, naturally timed storks (LMM, *F*_2,60.24_ = 8.890, *p* < 0.001, *n* = 4410; Tukey test, *p* < 0.001) and control storks (Tukey test, *p* < 0.01) left thermals at higher altitudes than delayed storks. Once leaving the thermals, delayed storks had a higher ground speed during gliding than naturally timed (figure 3*c*; LMM, *F*_2,65.28_ = 6.564, *p* < 0.005; Tukey test, *p* < 0.01, *n* = 4518) and control storks (Tukey test, *p* < 0.005), however, there was no significant difference in gliding airspeed (LMM, *F*_2,65.39_ = 0.371, *p* > 0.5, *n* = 4518) after correcting for differences in wind support (figure 3*d*; LMM, *F*_2,65.23_ = 8.194, *p* < 0.001, *n* = 4518), meaning delayed storks had better wind conditions and therefore glided faster. Yet, during gliding, naturally timed (LMM, *F*_2,65.41_ = 16.524, *p* < 0.001, *n* = 4518; Tukey test, *p* < 0.001) and control (Tukey test, *p* < 0.001) storks sunk faster than delayed storks.

We used ODBA as a measure for movement activity and thereby as a proxy for flapping activity. Groups differed in their flapping activity (figure 3*e*; LMM, *F*_2,65.41_ = 17.918, *p* < 0.001, *n* = 4373), specifically the delayed storks (mean ± sd: 4.21 ± 3.56; Tukey test, *p* < 0.001) and control storks (3.84 ± 3.69; Tukey test, *p* < 0.001) had a higher ODBA during migration flights within the segment than naturally timed storks (2.82 ± 2.81). These differences were mostly driven by low ODBA values for early naturally timed storks. Within the naturally timed group, there is a significant increase in ODBA over time (electronic supplementary material, figure S7; LMM, *F*_1,44.54_ = 64.349, *p* < 0.001, *n* = 2431). This corresponds to declining temperatures and boundary layer height as the season progresses (see electronic supplementary material, figure S8). To test if delayed storks and control storks had a higher ODBA than naturally timed storks because their time in an aviary deprived them of learning opportunities, we compared the northern and southern half of the migration segment. If learning would be important, we would expect to see improvements in ODBA over the course of the migration. There was no difference in ODBA between the northern and southern half of the migration segment for delayed and control storks (delayed: LMM, *F*_1,871.62_ = 0.126, *p* > 0.5, *n* = 881; control: LMM, *F*_1,1052.77_ = 3.007, *p* > 0.05, *n* = 1061). Within all groups, ODBA was higher during gliding than during climbing (naturally timed: LMM, *F*_1,1928.40_ = 6.731, *p* < 0.01, *n* = 2431; control: LMM, *F*_1,795.75_ = 36.134, *p* < 0.001, *n* = 1061; delayed: LMM, *F*_1,630.60_ = 27.037, *p* < 0.001, *n* = 881). A visual inspection of the data indicated that, during gliding segments, most flapping happened below 500 m above the ground (see electronic supplementary material, figure S9).

## 4. Discussion

We showed that the migration timing of juvenile white storks has not just important consequences for their short-term flight costs, but also affects their long-term migration and lifetime decisions. When migration timing is artificially delayed, some juvenile storks were able to conduct and complete their migration, but this delay had clear consequences for their overall migratory behaviour. Delayed juvenile storks exhibited a higher movement activity during flight, but also migrated faster and had a higher survival compared to regularly migrating storks. The immediate effects on flight performance were most likely caused by inferior thermalling conditions later in the season, however there was also a positive effect coming from more wind support. Finally, in addition to flight performance, the delayed migration timing also had longer-term effects by affecting migration destinations in the first and even in the subsequent year.

Migration timing affects migration speed at different scales. We found that delayed storks migrated faster by spending fewer days on stopovers than naturally timed and control storks. Previous studies on long-distance migrants also found a decline in stopover duration as the season progressed [55,56]. These findings suggest that later migrants may experience more pressure to reach their destination fast to, for example, avoid worsening weather conditions. Despite shorter daylengths for delayed storks, they did not migrate fewer hours per day than naturally timed storks. Enhanced wind support allowed delayed storks to cover distance faster on a daily basis and to glide faster between thermals than naturally timed and control storks. Although it is not clear whether an increase in wind support later in the season is a general pattern, our data show that delayed juveniles were able to fly under beneficial wind conditions later in the season.

White storks are soaring–gliding migrants that use thermals to save energy during migration. Naturally timed and control storks had higher climbing rates compared to the delayed storks, which is indicative of thermal properties. Delayed storks also left thermals at lower altitudes than storks in both other groups. Declining thermalling conditions (i.e. declining temperatures and boundary layer heights) could have caused the delayed storks to have a higher movement activity than naturally timed storks. Delayed and control storks had a higher ODBA than naturally timed storks, meaning that they flapped their wings more during migration flights. Most energetically costly flapping flight happened during gliding, and especially during gliding at low altitudes. This suggests that delayed storks used more flapping flight to avoid grounding while gliding between thermals [57]. Many of the delayed storks flew in proximity to each other during migration, which could have led to a similar flight performance. However, being in the same flock and experiencing the same conditions does not necessarily mean that individuals have the same flight performance [37]. In addition, a previous study on flight performance in white storks found that juveniles only reach the same flight performance of adults by the end of their first migration [31]. This suggests that the delayed juveniles in our study could have had a lower flight performance compared to the flock they were flying in. It may also be that artificially released storks lack flight experience, causing them to have a higher ODBA. The control group also migrated after an artificial release, however an improvement in flapping activity was not found for control and delayed storks. Although control storks were released earlier in the migration season, it may have caused them to migrate too late or under unfavourable conditions. Within the naturally timed group, ODBA increases as the season progresses. In addition, a recent study showed that later migrating adults have a higher ODBA than earlier migrants too [56]. Differences in ODBA might have been caused by differences in the physical environment, although we did not find evidence for this. Alternatively, differences in ODBA may have been caused by differences in the social environment; later migrating flocks consist of more experienced adults.

The consequences of migration timing go beyond immediate flight costs and migration duration. Despite their higher movement activity, delayed storks had a higher survival than naturally timed and control storks. Delayed juveniles could have been in better condition than regular juveniles since they were well fed during their time in the aviary. While control storks were also released from an aviary, this did not lead to an increased survival. The shorter migration distance of delayed storks could have contributed to their higher survival. While a reduced migration distance can enhance survival [38], complete residency can be detrimental as well. Part of our non-migrating delayed storks died from starvation during a harsh winter with a lot of snow in central Europe. This highlights why it is important for juvenile storks to migrate. Although they may rely partly on genetically inherited migration patterns, storks depend mostly on social information when establishing their migratory behaviour [35]. The motivation to depart on migration may be enhanced by the guidance of conspecifics. Some of our non-migrating delayed storks probably lacked social stimuli from other migrating storks. Instead, they encountered residents and therefore stayed locally. Due to differences in migration timing between, for example, populations and age groups, juveniles may benefit from migrating within a certain time window to match optimal social conditions. Similar to initial departure decisions, the decision to move between stopover sites, or to stay at an overwintering site, may also depend on social stimuli [5]. In white storks, the availability of social stimuli to depart from stopover sites likely reduces as the season progresses; more southern populations have an earlier migration onset [58] and later migrating adults shorten their migration distance with age [59]. The delayed storks that did migrate, migrated a shorter distance than regularly migrating storks. This suggests that migrating late deprives juveniles of the social environment that could have allowed them to continue their migration beyond adult overwintering sites. However, more studies are needed to reveal the relationship between population-level movements and juvenile decisions. Tracking entire populations is a promising approach to quantify the composition and dynamics of migratory flocks, as well as the connectivity of migratory populations [4]. Such a quantification of the social environment enables us to explore how social factors shape migratory behaviour, ranging from short-term decisions to lifetime development [60]. In addition, further experimental manipulations, such as translocation experiments, will allow us to disentangle the effects of the physical and social environment under ecologically relevant conditions.

Our results indicate that, when migrating, animals face a minimization trade-off between energy, time and risk [61]. This trade-off may differ between life stages due to the different requirements of the respective lifetime stage. For example, inexperienced individuals may migrate early to ensure they can rest longer during stopovers without losing social guidance. We suggest that, for storks, there could be a trade-off between migrating early under good thermalling conditions and migrating late with higher speeds through wind support. In addition, for social migrants, energy, time and risk may also be influenced by the social environment. Migrating late can affect the availability of social information, which can also influence fitness and survival. Dynamic and variable flocks with a high turnover may influence information transfer [62]. For example, a higher proportion of inexperienced individuals can lead to increased exploration [63] and even to the establishment of new overwintering sites [64]. Alternatively, exploration during early life may enable adults to establish new wintering areas by shortstopping (e.g. [65]). Our results indicate that delayed juveniles may not be able to compensate for the loss of exploration during their first migration, since delayed juveniles did not winter as far south as regular juveniles, even in their second year. This suggests that migration timing is crucial, not only to experience beneficial environmental and social conditions during migration, but also to enable flexible adjustments to changing conditions in the long term.

## Data Availability

The data used for this study are available through the Movebank Data Repository (https://www.datarepository.movebank.org/; naturally timed: [66]; control: [67]; delayed: [68]). The code used to process, analyse and visualize the data is available through Zenodo [69]. Supplementary figures and tables are provided in electronic supplementary material [70].
